# Gender difference in anxiety and related factors among adolescents

**DOI:** 10.3389/fpubh.2024.1410086

**Published:** 2025-01-03

**Authors:** Chengzhen Bao, Lili Han

**Affiliations:** Beijing Obstetrics and Gynecology Hospital, Capital Medical University, Beijing Maternal and Child Health Care Hospital, Beijing, China

**Keywords:** anxiety, adolescent, gender difference, risk factor, internet

## Abstract

**Background:**

Anxiety is widespread among adolescents, and research has shown that this condition can profoundly affect their mental, emotional, and physical well-being. The purpose of this study was to analyze gender differences in anxiety levels among adolescents and to explore the influencing factors and pathways.

**Methods:**

A total of 3601 adolescents were included in this study (age: 15.14±1.97 years; male: 48.76%). Gender, age, school category, grade, duration of sleep, duration on Internet, anxiety and several social factors were investigated by online questionnaire. Teachers were responsible for organizing students to fill out the questionnaire. The Generalized Anxiety Disorder (GAD-7) was applied to measure participants’ anxiety levels over the past 2 weeks. An Ordinal Logistic Regression measured risk factors of anxiety, while a path analysis was used to estimate the structural relationship between risk factors and anxiety.

**Results:**

The severity of anxiety in female was higher. Approaching graduation, lack of sleep, poor peer relationships, poor ability to complete tasks, and unwillingness to seek help when in a bad mood were risk factors for anxiety in both male and female adolescents. Among female, prolonged Internet access is a risk factor for anxiety. The fit indices for the modified models were appropriate (male: GFI=0.999, IFI=0.996, TLI=0.976, CFI=0.995, AGFI=0.990, RMSEA=0.021, SRMR=0.016; female: GFI=0.997, IFI=0.990, TLI=0.971, CFI=0.990, AGFI=0.990, RMSEA=0.020, SRMR=0.018).

**Conclusion:**

The female adolescents might have higher levels of anxiety, that academic stress, sleep, peer relationships, competence, and level of social support might be influence factors on anxiety in adolescents, and that “daily duration on Internet” might not be the risk factor in male adolescent.

## 1 Introduction

Anxiety was defined as an emotional state, with the subjectively experienced quality of fear as a closely related emotion, and it was categorized into state anxiety and trait anxiety. State anxiety could be interpreted as a transitory emotion, whereas trait anxiety was defined as an individual’s predisposition to respond (1). The COVID-19 pandemic could be seen as a massive traumatic experience. The traumatic nature of the COVID-19 pandemic was related not only to the physical health risks but also to the problems caused by the lockdown policy adopted by governments to slow the spread of the virus, such as life disruption (2, 3). This traumatic experience could trigger feelings of fear, restlessness, and a state of anxiety. As a result of the strict lockdown policy in China, the life disruption affected the mental health and wellbeing of adolescents and young adults, the level of anxiety sensitivity in adolescents became dramatically elevated (4, 5), and the percentage of adolescents with learning anxiety (33.7% vs. 56.4%) and somatic anxiety (13.9% vs. 40.7%) was increased (6).

As an inborn response to stimuli, anxiety can be characterized by increased heartbeat and respiration and accompanied by an acute sense of danger and dread (7). Anxiety has several negative effects on the health of adolescents. Among adolescents with congenital heart disease, anxiety may increase the risk for memory deficits (8). Voice disorders are associated with anxiety in adolescents (9). The higher levels of anxiety may relate to lower baroreflex sensitivity in young adults, which means that efficiency in short-term blood pressure regulation may decrease (10). Anxiety in childhood can progress to anxiety disorders (11), which may give rise to recurrent anxiety disorders during early adulthood much more easily (12) and result in more adverse psychosocial outcomes at 30 years old (13).

The risk factors for anxiety in adolescents contain several social factors such as lacking of peer acceptance and support (14), being bullied or excluded by others, not completing the whole of homework as required, being required to inform parents to communicate with teacher at school for the behaviors of their child (15), and high level of perceived competition in study (16). In addition, insufficient sleep time was an important factor associated with generalized anxiety disorder (17), and stress response plays an important role in the correlation between sleep difficulties and anxiety symptoms (18). Higher levels of anxiety were found among adolescents with more Internet addiction scores (19), and mindfulness moderates the relationship between mobile phone addiction and anxiety (20). In China, highly competitive senior high school and college admissions meant students who were in graduating classes should face greater academic pressure, which led to a higher risk of anxiety (21). Anxiety also has a genetic predisposition (22), and body mass index and drinking habits have been associated with anxiety (23).

Gao et al. found that the level of anxiety in female college students was higher than in male college students (23), and this study aims to identify if this gender difference also exists among adolescent aged 10–19 years? This study proposes two research hypotheses: (1) Chinese male and female adolescents had different levels of anxiety during the COVID-19 epidemic. (2) The factors influencing the anxiety levels of male and female adolescents and their pathways of action were different.

## 2 Methods

### 2.1 Participants

Data were collected from 16 districts in Beijing. Multi-stage sampling was applied to select participants: First, one middle school and one high school were selected, respectively, from each district by typical sampling method. Second, one class was selected in every grade, and the whole of the students in the class should be involved in the study. A total of 3,720 adolescents participated in this study, and 119 participants (3.20%) were excluded because of incomplete data. Finally, 3,601 subjects (male: 1,756, 48.76%; female: 1,845, 51.24%) were included. The average age of subjects was 15.14 years (SD: 1.97). Permission to conduct this study was obtained from the Beijing Maternal and Child Health Care Hospital. All of the participants were minors, so their guardians should sign informed consent before filling in the questionnaire.

### 2.2 Measures

Online survey was conducted during 1 April 2020 to 29 May 2020. Students were organized by their teachers to fill out the online questionnaire. Before the survey, teachers need to introduce the instructions for students to fill in the questionnaire, so as to reduce the rate of omission and mistake.

In the part of demographics, factors such as gender, grade, and school name were mentioned. Behavior-related factors contained sleeping (“sleep adequately” meant that people aged 10–12 years should sleep longer than 9 h and people aged 13–19 years should sleep longer than 8 h) (24) and duration on Internet (four options: 0.0–0.9 h/day; 1.0–1.9 h/day; 2.0–2.9 h/day; ≥3.0 h/day). Social factors consisted of “getting along with peer” (three options: “good,” “moderate,” and “bad”), “being bullied by peer” (three options: “never,” “sometimes,” and “always”), “ability to accomplish the tasks” (three options: “always as good as peer,” “sometimes as good as peer,” and “mostly worse than peer”), and “seeking help for bad moods” (being divided into “yes” and “no”).

The Generalized Anxiety Disorder-7 Item (GAD-7) was used as a screening tool of anxiety. As a self-report anxiety questionnaire, GAD-7 was developed to measure generalized anxiety disorder previously, and it had been found that reliability (corrected item-total correlations: 0.70 to 0.88, Cronbach’s *α* coefficients: 0.93–0.95) and validity (Spearman’s rank-order correlations with PHQ-9: 0.70–0.82) were satisfactory in Chinese Adolescents (25). The GAD-7 consisted of seven items, and the score of each item ranged from 0 to 3. The larger the score was, the greater risk of anxiety the subjects faced with. Scores of 15, 10, and 5 were cut-off points for severe, moderate, and mild anxiety, respectively (26).

### 2.3 Statistical analysis

The chi-square test was used to analyze the differences between male and female groups on distribution. Wilcoxon rank-sum test and Kruskal–Wallis test were used to compare the characteristics of the GAD-7 score. The risk factors of anxiety were analyzed by using ordinal logistic regression analysis. The conceptual models were prepared based on the findings from ordinal logistic regression analysis, and path analysis was used to estimate the structural relationship between risk factors and anxiety. Assumptions of the statistical analyses were tested and met. All statistical analyses were performed with SAS 9.2 and AMOS 24.0. Statistical significance was set as a *p*-value of <0.05. Good fit was indicated as follows: (1) GFI, IFI, and TLI > 0.95; (2) CFI and AGFI >0.90; (3) RMSEA and SRMR <0.08; (4) *p*-value (chi-square test) > 0.05; (5) *χ*^2^/*df* ≤ 3 (27, 28).

## 3 Results

### 3.1 Demographic characteristics

The median of the GAD-7 score was 2.00 (P_25_: 0.00; P_75_: 5.00), and female students (M: 2.00; P_25_: 0.00; P_75_: 6.00) were exposed to a higher risk of anxiety than male students (M: 1.00; P_25_: 0.00; P_75_: 5.00). Table 1 shows the characteristics of participants. Larger than half the proportion of male students were middle school students. The proportion of female students was larger in high school students. The percentages of graduating students were less than one-third both in male and female students, and the ratio of graduating students in male students was larger than that of female students (*p* < 0.05). Compared with male students, the proportion of female students who could keep sleeping adequately was less (*p* < 0.05). The majority of subjects could get along well with peer, and the percentage of subjects who reported “moderate” was less in male students. In comparison with female students, both the ratios of subjects who had never been bullied by peer and who were always bullied were larger in male students. The difference in the ability to accomplish the tasks between male and female students was statistically significant, and the larger proportion of male students reported “always as good as peer.” The percentage of subjects who would seek help for bad moods was larger in female students (*p* < 0.05). Whichever grade of anxiety (mild, moderate, and severe), the problems were worse in female students.

**Table 1:** Characteristics of distribution among the adolescents by gender.

Variables	Total		Male		Female		*c* ^2^
	*N*	%	*N*	%	*N*	%	
School							8.43^*^
Middle school	1,783	49.51	913	51.99	870	47.15	
High school	1,818	50.49	843	48.01	975	52.85	
Graduating student							5.16^*^
Yes	1,048	29.10	542	30.87	506	27.43	
No	2,553	70.90	1,214	69.13	1,339	72.57	
Keeping sleeping adequately							22.98^*^
Yes	1,278	35.49	692	39.41	586	31.76	
No	2,323	64.51	1,064	60.59	1,259	68.24	
Daily duration on Internet							4.32
0.0–0.9 h	1,206	33.49	561	31.95	645	34.96	
1.0–1.9 h	989	27.46	484	27.56	505	27.37	
2.0–2.9 h	601	16.69	302	17.20	299	16.21	
≥3.0 h	805	22.35	409	23.29	396	21.46	
Getting along with peer							24.55^*^
Good	2,677	74.34	1,366	77.79	1,311	71.06	
Moderate	799	22.19	328	18.68	471	25.53	
Bad	125	3.47	62	3.53	63	3.41	
Being bullied by peer							17.15^*^
Never	2,940	81.64	1,435	81.72	1,505	81.57	
Sometimes	595	16.52	273	15.55	322	17.45	
Always	66	1.83	48	2.73	18	0.98	
Ability to accomplish the tasks							17.07^*^
Always as good as peer	2,743	76.17	1,382	78.70	1,361	73.77	
Sometimes as good as peer	797	22.13	339	19.31	458	24.82	
Mostly worse than peer	61	1.69	35	1.99	26	1.41	
Seeking help for bad moods							8.91^*^
Yes	2,186	82.43	937	79.95	1,249	84.39	
No	466	17.57	235	20.05	231	15.61	
Anxiety (GAD-7 score)							25.26^*^
None (0–4)	2,506	69.59	1,290	73.46	1,216	65.91	
Mild (5–9)	782	21.72	337	19.19	445	24.12	
Moderate (10–14)	193	5.36	76	4.33	117	6.34	
Severe (≥ 15)	120	3.33	53	3.02	67	3.63	

Analyzing the differences between male and female students by the chi-square test; ^*^p < 0.05.

### 3.2 The distribution of the GAD-7 score with different characteristics

The distribution of the GAD-7 score with different characteristics is shown in Table 2. High school students or graduating students are faced with sever anxiety significantly both in male and female students. The differences in the distributions of behavior-related factors (e.g., keeping sleeping adequately and daily duration on Internet) and social factors (e.g., getting along with peer, being bullied by peer, ability to accomplish the tasks, and seeking help for bad moods) were statistically significant in male and female students, respectively. Subjects who could keep sleeping adequately or take less time on Internet got lower scores on GAD-7. No matter male or female students, those who reported getting along well with peer, or having never been bullied by peer, or always accomplishing tasks as good as peer or being willing to seek help for bad moods might keep lower levels of anxiety. To contrast, there were larger gaps on the medians of different groups of social factors, especially “getting along with peer,” “being bullied by peer,” and “ability to accomplish the tasks.” In the majority of groups, the medians of GAD-7 scores in female students were larger.

**Table 2:** Distribution of the GAD-7 score with different characteristics.

Variables	Male (*N* = 1,756)			Female (*N* = 1,845)		
	Median (*P*_25_, *P*_75_)	Mean ± SD	*Z*/*c*^2^	Median (*P*_25_, *P*_75_)	Mean ± SD	*Z*/*c*^2^
School			3.89^*^			−4.65^*^
Middle school	1.00 (0.00, 4.00)	2.70 ± 4.09		2.00 (0.00, 5.00)	3.57 ± 4.66	
High school	2.00 (0.00, 6.00)	3.39 ± 4.49		3.00 (1.00, 6.00)	4.00 ± 4.15	
Graduating student			6.63^*^			5.94^*^
Yes	2.00 (0.00, 7.00)	4.07 ± 4.97		4.00 (1.00, 7.00)	4.48 ± 4.39	
No	1.00 (0.00, 4.00)	2.57 ± 3.87		2.00 (0.00, 5.00)	3.53 ± 4.38	
Keeping sleeping adequately			−6.34^*^			−9.23^*^
Yes	1.00 (0.00, 3.00)	2.32 ± 3.81		1.00 (0.00, 4.00)	2.63 ± 3.74	
No	2.00 (0.00, 6.00)	3.50 ± 4.53		3.00 (1.00, 7.00)	4.34 ± 4.58	
Daily duration on Internet			18.46^*^			43.84^*^
0.0–0.9 h	1.00 (0.00, 4.00)	2.49 ± 3.82		2.00 (0.00, 5.00)	3.15 ± 4.03	
1.0–1.9 h	1.00 (0.00, 5.00)	2.90 ± 4.03		2.00 (0.00, 5.00)	3.56 ± 3.94	
2.0–2.9 h	1.00 (0.00, 6.00)	3.30 ± 4.61		3.00 (0.00, 7.00)	3.95 ± 4.40	
≥3.0 h	2.00 (0.00, 7.00)	3.73 ± 4.84		4.00 (1.00, 7.00)	5.03 ± 5.22	
Getting along with peer			415.31^*^			397.93^*^
Good	0.00 (0.00, 3.00)	1.94 ± 3.26		1.00 (0.00, 4.00)	2.55 ± 3.43	
Moderate	6.00 (3.00, 7.00)	5.91 ± 4.15		6.00 (3.00, 8.00)	6.33 ± 4.52	
Bad	13.00 (6.00, 20.00)	11.84 ± 7.19		11.00 (5.00, 15.00)	10.68 ± 6.52	
Being bullied by peer			294.71^*^			276.62^*^
Never	1.00 (0.00, 3.00)	2.19 ± 3.46		2.00 (0.00, 5.00)	2.98 ± 3.75	
Sometimes	6.00 (2.00, 7.00)	5.93 ± 4.74		7.00 (4.00, 9.00)	7.20 ± 5.07	
Always	11.50 (7.00, 18.50)	11.79 ± 6.69		12.00 (8.00, 15.00)	11.22 ± 5.96	
Ability to accomplish the tasks			300.16^*^			311.19^*^
Always as good as peer	1.00 (0.00, 3.00)	2.13 ± 3.44		1.00 (0.00, 4.00)	2.76 ± 3.58	
Sometimes as good as peer	6.00 (2.00, 7.00)	5.64 ± 4.58		6.00 (3.00, 8.00)	6.34 ± 4.74	
Mostly worse than peer	14.00 (9.00, 21.00)	13.49 ± 7.08		14.00 (7.00, 19.00)	13.12 ± 7.36	
Seeking help for bad moods			8.15^*^			8.31^*^
Yes	2.00 (0.00, 5.00)	3.18 ± 4.22		3.00 (0.00, 6.00)	3.84 ± 4.16	
No	5.00 (1.00, 9.00)	6.02 ± 5.53		6.00 (2.00, 10.00)	6.75 ± 5.51	

Analyzing the differences by Wilcoxon rank-sum test or Kruskal–Wallis test; ^*^p < 0.05.

### 3.3 The risk factors of anxiety

The risk factors for anxiety were analyzed by gender (Table 3), which were a little different between male and female students. In the results, anxiety of both genders was statistically associated with being studying in graduating class or not. In addition, in whichever gender adolescents who could not keep sleeping adequately might face with greater risk of anxiety. For female students, longer time taken on Internet meant severe anxiety who might be suffering from, which was different with male students. Among male and female students, getting along badly with peers might become one of the reasons for anxiety. The adolescents who had been bullied by peer seemed to be more prone to anxiety regardless of gender. Moreover, the weaker ability the adolescents had to accomplish the tasks meant the higher risk of anxiety in male and female students. In addition, it was a risk factor for anxiety in both genders when he or she was unwilling to seek help for bad moods.

**Table 3:** Risk factors of anxiety among the adolescents by gender.

Variables	Male (*N* = 1,756)			Female (*N* = 1,845)		
	*β*	*p*	OR (95%CI)	*β*	*p*	OR (95%CI)
Graduating student (Ref. = No)	0.39	<0.01	1.47 (1.12, 1.94)	0.40	<0.01	1.49 (1.17, 1.90)
Keeping sleeping adequately (Ref. = Yes)	0.29	<0.05	1.34 (1.00, 1.79)	0.57	<0.01	1.78 (1.37, 2.31)
Daily duration on Internet (Ref. = 0.0 ~ 0.9 h)						
1.0–1.9 h	–	–	–	0.35	<0.05	1.43 (1.06, 1.91)
2.0–2.9 h	–	–	–	0.44	<0.05	1.55 (1.11, 2.18)
≥3.0 h	–	–	–	0.79	<0.01	2.21 (1.63, 2.99)
Getting along with peer (Ref. = Good)						
Moderate	1.15	<0.01	3.17 (2.22, 4.50)	0.94	<0.01	2.55 (1.93, 3.38)
Bad	1.82	<0.01	6.16 (3.16, 12.02)	1.97	<0.01	7.20 (4.05, 12.80)
Being bullied by peer (Ref. = Never)						
Sometimes	0.52	<0.01	1.68 (1.16, 2.42)	0.61	<0.01	1.84 (1.36, 2.48)
Always	1.59	<0.01	4.89 (2.29, 10.46)	1.11	<0.05	3.03 (1.08, 8.52)
Ability to accomplish the tasks (Ref. = Always as good as peer)						
Sometimes as good as peer	0.58	<0.01	1.79 (1.28, 2.52)	0.64	<0.01	1.91 (1.46, 2.50)
Mostly worse than peer	1.86	<0.01	6.40 (2.63, 15.58)	1.82	<0.01	6.16 (2.69, 14.09)
Seeking help for bad moods (Ref. = Yes)	1.12	<0.01	3.06 (2.26, 4.13)	0.86	<0.01	2.36 (1.78, 3.14)

### 3.4 The conceptual models of anxiety

**Figure 1:**
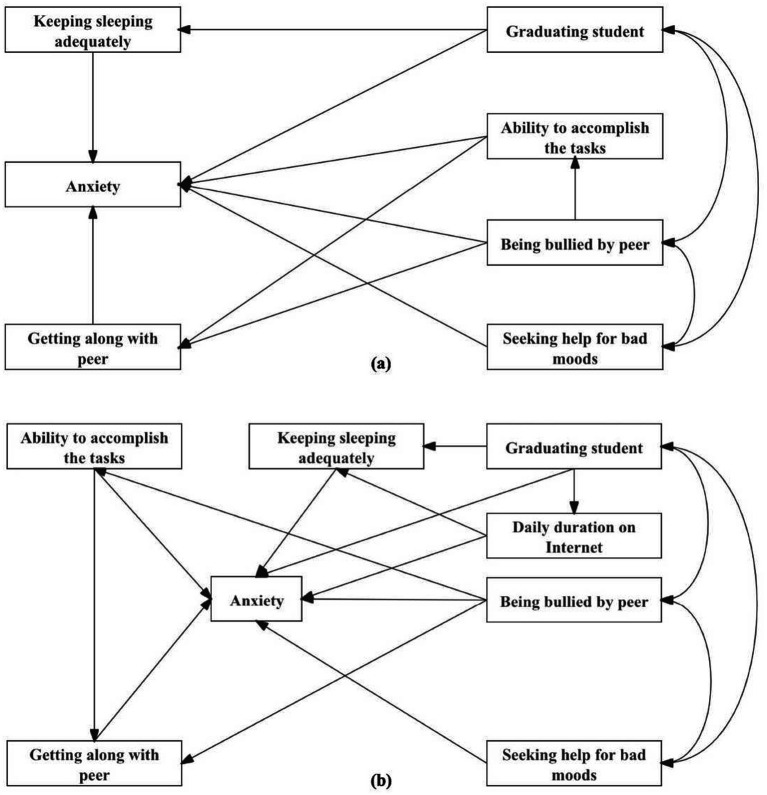
Priori hypothesized models between risk factors and anxiety for male **(A)** and female **(B)** adolescents.

**Figure 2:**
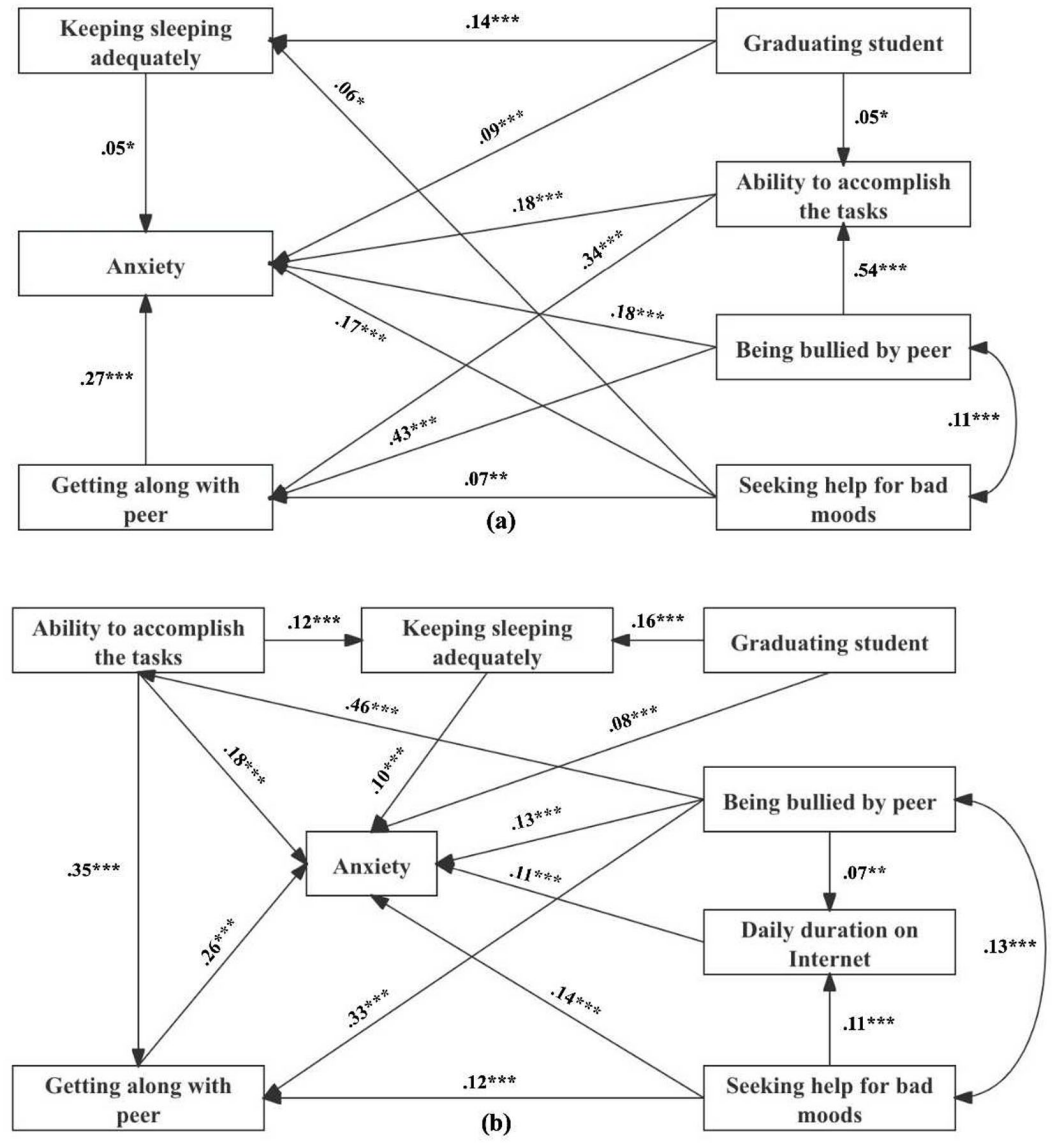
Path analysis models between risk factors and anxiety for male **(A)** and female **(B)** adolescents. *p < 0.05; **p < 0.01; ***p < 0.001.

The priori hypothesized models are shown in Figure 1, and the fit indices of two models were poor (male: TLI = 0.878, *χ*^2^ = 29.386, *p* < 0.05, *χ*^2^/*df* = 3.673; female: IFI = 0.871, TLI = 0.689, CFI = 0.867, *χ*^2^ = 84.544, *p* < 0.05, *χ*^2^/*df* = 7.045). The fit indices for the modified models were appropriate (male: GFI = 0.999, IFI = 0.996, TLI = 0.976, CFI = 0.995, AGFI = 0.990, RMSEA = 0.021, SRMR = 0.016, *χ*^2^ = 6.143, *p* > 0.05, *χ*^2^/*df* = 1.536; female: GFI = 0.997, IFI = 0.990, TLI = 0.971, CFI = 0.990, AGFI = 0.990, RMSEA = 0.020, SRMR = 0.018, *χ*^2^ = 15.666, *p* > 0.05, *χ*^2^/*df* = 1.567), and differences on models could be found between male and female students (Figure 2). For male students, the model consisted of four endogenous (keeping sleeping adequately, getting along with peer, ability to accomplish the tasks, and anxiety) and three exogenous (graduating student, being bullied by peer, and seeking help for bad moods) variables, which were not completely same in female students and another endogenous variable named daily duration on Internet should be included. As Table 4 shows, the direct, indirect, and total effects of the variables examined in the modified models were various from different genders. With regard to total effects, being bullied by peer had the most effect on anxiety in male and female students, whereas this effect was stronger for male than female students.

**Table 4:** Results of path analysis: the direct, indirect, and total effects of the variables in the conceptual models for male and female adolescents.

Variables	Male			Female		
	Direct effects	Indirect effects	Total effects	Direct effects	Indirect effects	Total effects
Graduating student → Anxiety	0.086	0.021	0.107	0.077	0.015	0.092
Keeping sleeping adequately → Anxiety	0.052	–	0.052	0.096	–	0.096
Daily duration on Internet → Anxiety	–	–	–	0.110	–	0.110
Getting along with peer → Anxiety	0.267	–	0.267	0.262	–	0.262
Being bullied by peer → Anxiety	0.178	0.259	0.437	0.132	0.223	0.355
Ability to accomplish the tasks → Anxiety	0.179	0.090	0.269	0.180	0.103	0.283
Seeking help for bad moods → Anxiety	0.171	0.021	0.192	0.138	0.042	0.180
Graduating student → Keeping sleeping adequately	0.138	–	0.138	0.155	–	0.155
Graduating student → Getting along with peer	–	0.017	0.017	–	–	–
Graduating student → Ability to accomplish the tasks	0.050	–	0.050	–	–	–
Being bullied by peer → Keeping sleeping adequately	–	–	–	–	0.056	0.056
Being bullied by peer → Daily duration on Internet	–	–	–	0.071	–	0.071
Being bullied by peer → Getting along with peer	0.426	0.182	0.608	0.326	0.160	0.486
Being bullied by peer → Ability to accomplish the tasks	0.540	–	0.540	0.460	–	0.460
Ability to accomplish the tasks → Keeping sleeping adequately	–	–	–	0.121	–	0.121
Ability to accomplish the tasks → Getting along with peer	0.337	–	0.337	0.347	–	0.347
Seeking help for bad moods → Daily duration on Internet	–	–	–	0.105	–	0.105
Seeking help for bad moods → Keeping sleeping adequately	0.060	–	0.060	–	–	–
Seeking help for bad moods → Getting along with peer	0.065	–	0.065	0.115	–	0.115

## 4 Discussion

The present study analyzed the differences on degrees of anxiety and explored the predictors and conceptual models of anxiety among adolescents by gender. Higher GAD-7 scores were related to greater pressure of study (such as “graduating student” in this study), more inadequate sleep, worse peer relationship, poorer ability to accomplish the tasks, and less social support in terms of mood regulation in both genders. In addition, a particular risk factor of anxiety for female students in this study was daily duration on Internet. The above findings implied that prevention and intervention for adolescent anxiety should take into account gender differences, use more targeted measures, and provide more support for adolescents with risk factors for anxiety, such as more frequent screening and group counseling.

The unsynchronized development of anxiety-relevant brain functional systems resulted in the peak period for the incidence of anxiety disorders in adolescence (29). The development of anxiety symptoms was found, that is, anxiety symptoms would decrease during early adolescence and then subsequently increase from middle-to-late adolescence (30). In this study, we found that female adolescents might suffer from severe anxiety than male adolescents during the COVID-19 pandemic, which was similar to other research studies (31). On the physical side, the change of endocrine during the menstrual cycle might have an influence on emotional states, and anxiety was one of the prevalent symptoms among female adolescents with premenstrual syndrome (32). Female adolescents displayed a positive relationship between anxiety and the error-related negativity amplitude, and this neurocognitive mechanism might drive the gender difference in anxiety (33). On the psychological side, it was found that girls in high school experienced greater academic stress than boys (34), which might cause the degree of anxiety to increase. Moreover, previous studies mentioned that low self-esteem was risk factor of anxiety (35) and female adolescents exhibited lower levels of self-esteem (36), which meant that higher levels of anxiety might be found in female adolescents.

In a previous study, a moderate positive association was found between Internet addiction and social anxiety (37). In some studies, those who were classified as having high level of Internet addiction or heavy Internet use featured significantly severer symptoms of anxiety or higher level of anxiety (38–40), which meant that excessive Internet use might increase the risk of anxiety. During the COVID-19 pandemic, schools adopted online courses to avoid unnecessary crowd gatherings. The sudden change in the mode of teaching and learning was a challenge for students. Students with poor adaptability might experience a decrease in the grade and an increase in academic stress and anxiety (41). In online courses, students could not easily feel teachers’ support and emotional feedback, which was more likely to trigger anxiety (42), and screen exposure as a result of online learning increased the incidence and severity of anxiety-related disorders (e.g., tension-type headache) (43–45). In this study, “daily duration on Internet” was a risk factor of anxiety only in female students, which might be caused by poorer adaptability of long-time Internet use (46).

Adolescents, both male and female, who were graduating students might be more susceptible to anxiety based on the findings of this study, which might result from more academic pressure they had (47). In this study, short sleep duration every night might lead to an increasing risk of anxiety among adolescents, and similar conclusions also had been reported in a previous study (48). Pickering et al. found that poor peer relationship was significantly associated with increased social anxiety (14). In this study, poorer relationships were mainly characterized by being bad at getting along with peer and always being bullied by peer. Having poorer ability than peer to accomplish the tasks resulted in the severe anxiety of adolescents in this study might be due to increasing stress in daily life (49). Help for bad moods could be regarded as social support and it could significantly moderate the relationship between stress and anxiety (50), so as we found in this study being unwilling to seek help for bad moods might have a negative effect on the mental health of adolescents.

The conclusions of this study should be considered in light of limitations. First, a typical sampling method was used in the stages of school selection and class selection, so the representativeness of sample was unsatisfactory. Second, anxiety was not a hospital-based diagnosis in this study, so the results cannot be generalized to clinical interventions and therapeutics. Finally, the majority of questions were qualitative questions (e.g., how do you get along with peer), although qualitative questions were easier to answer for students, dose–response relationships could not be analyzed based on qualitative questions.

## 5 Conclusion

At the beginning of this study, we asked whether there were gender differences in the levels of anxiety among Chinese adolescents. It was found that the severity of anxiety in female students was higher, which might be caused by several physical and psychological reasons. After being grouped by gender and other features (e.g., demographic factors, behavior-related factors, and social factors), the medians of GAD-7 scores in female students were almost always larger than those in male students in every sub-group. Based on the findings of multiple-factor analyses, “daily duration on Internet” might be the only risk factor of anxiety that could be found in female adolescents rather than male adolescents, and other risk factors related to academic pressure, sleeping, peer relationship, ability, and social support might be found in both genders. To prevent anxiety among adolescents, gender-related strategies should be carried out in the future.

## Data Availability

The data analyzed in this study is subject to the following licenses/restrictions: the data that has been used is confidential. Requests to access these datasets should be directed to hanll2004@ccmu.edu.cn.
